# Regional differences and determinants of self-rated health in a lower middle income rural Society of China

**DOI:** 10.1186/s12939-018-0875-0

**Published:** 2018-11-08

**Authors:** Lidan Wang, Weizhen Dong, Yunqing Ou, Shuting Chen, Jingjing Chen, Qicheng Jiang

**Affiliations:** 1 School of Health Management, Anhui Medical University, No.81, Mei Shan Road, Hefei, 230032 Anhui China; 2 Anhui No.2 Provincial People’s Hospital, NO.1868, Dangshan Road, Hefei, 230041 Anhui China; 3 Department of Sociology and Legal Studies, University of Waterloo, 200 University Avenue West, Waterloo, ON N2L 3G1 Canada; 4 Anhui Provincial Cancer Hospital, No. 107, Lake Road, Hefei, 230031 Anhui China; 5 Children’s Hospital of Nanjing Medical University, No.72,Guang Zhou Road, Nanjing, 210008 Jiangsu China; 6 School of Public Health, Anhui Medical University, No.81, Mei Shan Road, Hefei, 230032 Anhui China

**Keywords:** Social determinants, Health status, Regional differences, Rural China

## Abstract

**Objective:**

Self-rated health represents a reliable and important health measure related to general health and quality of life. This study aimed to identify the differences of health states of rural residents in a lower middle income setting in China and its associated factors.

**Methods:**

A descriptive study of a stratified random sample of 3870 individuals was conducted in rural Anhui during 2015. We investigated the influence of five independent variables: individual demographic characteristics, family factors, social capital traits, physical health conditions and healthy lifestyle habits of participants who self-related their health as good. A chi-square test and ordinal logistic regression analyses were used to identify the relationship of these variables and self-rated health.

**Results:**

The study found that respondents who negatively rated their health often were female, elderly, poor, lived alone, had low levels of education, inadequate social support, poor physical health, used healthcare services and lived in the lower economic regions. We found no significant correlations between self-rated health and employment, marital status, medical insurance, or exercise frequency. Surprisingly, smoking and drinking also seemed to be unrelated to poor self-reported health.

**Conclusion:**

Health differences based on region were apparent in rural China. We highlighted the possible impacts of income, age, physical health, education, advanced age, and social support on health. The results from this study could inform the delivery of appropriate health and social healthcare interventions to promote rural residents’ health and quality of life.

## Introduction

Self-rated health (SRH) is widely known as a general perception of individual health status [1, 2], and a key indicator to measure health in population-based studies [3]. SRH has been approved by organizations such as the World Health Organization [4], because it provides a simpler [5], less expensive [6], more precise, and more objective measure of health compared to clinical evaluations [7]. Furthermore, the validity of SRH measure of self-report health has been firmly established in population studies.

The profound understanding of predictors of good SRH for persons in the general population has increased considerably in recent years. Initially an exploration of SRH began with personal demographic characteristics (e.g. age, gender, marital status, education, income) [8, 9], individual healthy lifestyle habits (e.g. smoking, poor nutrition, lack of exercise, drinking, awareness of dietary guidelines) [2, 10], and the state of physical and mental health (e.g. functional capacity, family history of hereditary disease, depression, anxiety, work stress) [11, 12]. Investigators have broadened the field of study to include broad-social concerns (social capita, social support, neighbor quality) [13–15] and factors related to living conditions (e.g. housing conditions, material conditions, level of crime, regional differences, economic crisis, psychosocial conditions) [10, 15, 16].

In general, domestic studies have focused on the elderly or urban residents [17]. However, the use of SRH measures of the health of rural residents has lagged, presumably because of the assumption that the characteristics of socioeconomic, healthy lifestyle habits, physical health and utilization of healthcare services, prove widely divergent in the large area that rural China encompasses. Interestingly, disparities in heath statuses among different regions in China have elicited a growing concern for the health challenges of rural populations. How do rural residents view their own health? What shapes the regional discrepancies among rural residents’ SRH levels? To address these questions demands an examination of the factors associated with SRH. A better understanding of the impact that social factors have on rural residents’ SRH would permit the development of improved intervention strategies to assure enhanced quality of health in later life.

The present study was conducted with rural residents of Anhui Province to identify health conditions among the population as well as the regional disparities that exist. Factors that influenced rural residents’ SRH were examined to determine if they were different from those in other regions and to provide data that could contribute to the improvement of health conditions in rural area and lower middle income societies.

## Methods

### Ethics statement

The study was approved by the Anhui Medical University Research Ethics Committee. Each participant received an explanation of the significance of the study and a form that outlined the protection of personal information from trained data collectors. All participants provided written informed consent.

### Data collection

Anhui province is located in the east of China. The province boasted 61.436 million permanent residents in 2015 (ranking 8th among 34). The Gross Domestic Product (GDP) of Anhui was 5808 dollars per capita in 2015, ranking 25th among 34. The Percentage of healthcare to consumer expenditures in rural areas was 8.99% [18]. The study included Anhui rural residents over the age of 15 years old as research subjects. Under a multi-stage stratified random sampling method, the survey randomly selected 3 of the 16 municipalities, representing three geographical areas (southern, central and northern regions). In each 3 regions, 2 counties were selected by economic level (wealthier and poorer). Three villages were randomly selected from each county. A total of 18 villages in 3 regions were selected. All the permanent and available residents from farming households were invited to participate in the survey. In this study, “permanent residents” were defined as those who spent more than 6 months out of the year at the registered place of residence. From June to August 2015, the survey was conducted in the subjects’ homes. The questions covered demographic and socio-economic information on individuals and households, socio-economic status, physical health, and healthy lifestyle habits.

A total of 3870 valid questionnaires were collected from the 3 regions. Additional information on the geography and economic profiles of the regions was collected from local statistics bureaus (Table 1).

**Table 1:** Socioeconomic characteristics of sampled regions (2015)

Variable	Southern region	Central region	Northern region	Total Anhui
Population/square kilometers	459	420	652	438
Per capita GDP ($)	13,001	2968	1769	5808
Per capita disposable income of rural household($)	2835	2298	1482	1738
Numbers of medical practitioners /1000 population	2.07	2.28	1.07	1.55
Utilization rate of city hospital beds (%)	82.44	86.36	86.66	84.97
Utilization rate of township hospital beds (%)	26.65	51.13	80.70	60.64

### Definition of key study variables

#### Dependent variable

In the study, SRH status was assessed using the single-item question “What would you say that your overall physician heath is?” Participants were asked to complete the sentence with a number on a scale of 0(poor) to 100(excellent). Additionally, participants were asked to provide a score that captures their health on that day they completed the survey. The scores were divided into 4 quartiles from lowest (scores of less than 60.00) to highest (scores higher than 90.00).

#### Independent variables

The independent variables included were as follows: individual demography characteristics, family factors, social capital traits, physical health, and healthy lifestyle habits. Individual demography characteristics included gender, age, education and employment status. Family factors included marital status, annual household income per capita, and family composition. Annual household income per capita was divided into 4 quartiles from lowest to highest. Indicators of social capital traits included medical insurance and social support. In urban China, there were two main forms of medical insurance during the time of the study, medical insurance for urban employees and medical insurance for urban residents respectively. In addition, the New Rural Cooperative Medical System (NRCM) was introduced in 2003 and extended to almost all rural residents by 2010. The Social Support Rating Scale (SSRS) was used to measure social support, including the three dimensions of objective support, subjective support, and utilization of social resources. The scores were grouped from lowest to highest.

Physical health conditions included clinic visits (in the past two weeks), hospitalization (during the past year) and the presence of common chronic disease (such as hypertension disease, diabetes disease, cardiovascular and cerebrovascular diseases) diagnosed by doctor. Healthy lifestyle habits included outdoor exercise, as well as the absence of smoking or drinking. Residence was divided geographically into southern, central, and northern regions of Anhui province. Definitions of the variables appears in Table 2.

**Table 2:** Descriptive characteristics of participants in different districts (%)

Variable		Southern region (*n* = 1245)	Central region (*n* = 1537)	Northern region (*n* = 1088)	Total Anhui (*n* = 3870)
Gender^**^	Male	42.62	40.45	36.76	40.27
	Female	57.38	59.55	63.24	59.73
Age(y)^**^	15~	25.31	28.11	19.58	24.60
	45~	29.02	28.27	26.29	28.01
	55~	22.58	21.29	24.63	22.74
	65~	23.10	22.33	29.50	24.65
Education^**^	No education	42.64	38.63	52.02	43.99
	Primary school	26.04	27.95	25.55	26.52
	Middle school or higher	31.32	33.41	22.43	29.49
Employment status^**^	Subsistence farmers	68.50	57.19	83.49	69.13
	Others	31.50	42.81	16.51	30.87
Marital statue	Married	88.29	85.70	85.37	86.64
	Others	11.71	14.30	14.63	13.36
Income^*^	Q1 (~ 25%)	24.06	24.75	24.36	24.37
	Q2	24.96	25.22	24.74	24.99
	Q3	27.24	24.34	23.76	25.38
	Q4(75%~)	23.73	25.69	27.15	25.26
Household composition^**^	Parents and children	26.22	30.44	19.85	25.79
	Three generations	27.33	19.52	15.17	21.40
	Elderly and children	8.46	5.78	13.97	9.15
	Only elderly	37.54	43.78	50.74	43.26
	Others	0.46	0.48	0.28	0.41
Medical insurance^**^	NRCMs	96.16	88.51	98.81	94.44
	Other medical insurances	2.86	7.87	0.74	3.88
	No medical insurance	0.98	3.61	0.46	1.68
Social support^**^	Q1 (~ 25%)	25.70	24.74	47.98	31.65
	Q2	21.21	21.61	21.23	21.34
	Q3	25.11	27.39	15.90	23.26
	Q4(75%~)	27.98	26.27	14.89	23.75
Chronic disease^**^	No	48.86	54.49	42.10	48.77
	Yes	51.14	45.51	57.90	51.23
Clinic visit^**^	No	61.99	57.98	40.00	54.54
	Yes	38.01	42.02	60.00	45.46
Hospitalization	No	11.05	11.66	22.50	14.46
	Yes	88.95	88.34	77.50	85.54
Active exercise^**^	No	80.29	68.40	73.40	74.52
	Yes	19.71	31.60	26.60	25.46
Smoke^**^	No	78.09	77.98	83.61	79.60
	Yes	21.91	22.02	16.39	20.40
Drink^**^	No	76.58	77.51	86.21	79.59
	< 3 times/week	8.56	7.31	5.15	7.24
	≥3 times/week	14.77	15.18	8.64	13.18

^a^Other medical insurance: basic medical insurance for urban and rural residents, commercial health insurance

^b^Below the median score

^c^Equal to or higher than the median score

^*^Significant difference among respondents from 3 regions at *P* < 0.05 level

^**^Significant difference among respondents from 3 regions respondents at *P* < 0.01 level

#### Active exercise

Outdoor exercise was assessed with the following item: “Including all types of outdoor exercise, how many times during one week did you exercise outdoors for more than 30 minutes in the last 12 months, excluding everyday activities and working in the field?” Those who reported exercise on one or more occasions were categorized as “Yes;” all other participant response were labeled “No.”

#### Smoking status

Smoking status was assessed with the following item: What is your history of cigarette smoking? Possible responses were: (1) I have never smoked; (2) I only smoked on one or two occasions; (3) I smoked regularly (at least once per day), but have quit; (4) I smoked, but not every day; and (5) I smoked every day. Participants selecting responses 1, 2 or 3 were labelled “not a smoker,” participants selecting responses 4 or 5 were defined “Smoker.”

#### Drinking status

Level of drinking was determined by the following question: How many times in any given week did you drink in the last year? Possible responses were: (1) I have never drunk; (2) I used to drink but quit at least 6 months ago; (3) I only drank on one or two occasions; (4) I drank less than 3 times a week, and (5) I drank at least 3 times a week. Participants who chose responses 1, 2 or 3 were defined as “tot a drinker”, participants who chose responses 4 were defined as “< 3 times/week,” and respondents who selected responses 5 were defined as “≥3 times/week.”

#### Statistical analysis

Data analysis was conducted using SPSS 23.0. Differential testing was conducted using a chi-square test. A series of ordinal logistic regression models were performed to determine associations between SRH and the independent variables. A value of *P* < 0.05 was considered to be significant.

## Results

### Characteristics of the sampled regions and participants

The central region was close to the provincial capital, and the per capita. The GDP per capita was $2986 in 2015. The southern region showed the highest economic level (per capita GDP, $13001) and the highest disposable income ($2835 per capita). Residents of the northern plain lived closer to each other (652 persons per km^2^), the lowest economic level and the fewest number of medical practitioners (1.07/1000 residents). However, the utilization rate of city and township hospital beds were highest in the northern region (86.66% and 80.70%, respectively). The disparity of utilization of township hospitals beds among the 3 regions was larger than that of city hospitals (Table 1).

As shown in Table 2, the demographic composition of the 3 regions indicated no statistically significant difference in terms of marital status (*P* > 0.05), but all of the demographic social traits were statistically distinct. There were more residents who were female (63.24%), over 65 years of age (29.50%), without education (52.02%), subsistence farmers (83.49%), and elderly living alone (50.74%) in the northern region than elsewhere. Most of the sampled households used by NRCM coverage, especially in the northern region (98.81%). Northern residents demonstrated the highest social support (47.98%), chronic illnesses (57.90%), injury or illness within the past two weeks (60.00%), hospitalizations within the past year (77.50%), outdoor exercise (25.46%), and abstinence from alcohol (86.21%). However, few northern residents smoked (16.39%).

### Individual and family traits and health

In general, there were statistically significant differences with regard to the association between SRH and demographic traits, with the exception of gender, medical insurance and active exercise (Table 3). The SRH levels of male and female did not prove significantly different between the central and northern regions.

**Table 3:** Percentage of self-rated health among different groups (%)

Characteristic		Southern region				Central region				Northern region			
		Poorest	Poor	Good	Best	Poorest	Poor	Good	Best	Poorest	Poor	Good	Best
Gender	Male	21.27^**^	20.48	26.84	31.41	*25.19*	*20.00*	*25.19*	*29.62*	*46.25*	*20.75*	*16.50*	*16.50*
	Female	27.09	22.37	27.76	22.78	26.08	22.11	25.06	26.76	48.98	21.51	15.55	13.95
Age(y)	15~	10.86^**^	16.00	31.14	42.00	10.54^**^	16.97	27.76	44.73	25.35^**^	16.90	22.54	35.21
	45~	18.18	23.58	28.13	30.11	21.75	25.56	25.34	27.35	42.66	21.33	19.58	16.43
	55~	35.85	24.53	23.40	16.23	33.14	18.44	25.65	22.77	51.12	24.25	14.55	10.07
	65~	39.93	23.38	25.54	11.15	40.00	23.10	21.41	15.49	65.11	21.50	9.35	4.05
Education	No education	35.34^**^	24.74	26.40	13.51	36.64^**^	21.37	23.51	18.47	57.07^**^	21.55	12.90	8.48
	Primary school	28.45	22.99	25.86	22.70	22.50	24.25	24.50	28.75	44.24	22.30	16.55	16.91
	Middle school or higher	9.38	16.83	29.81	43.99	13.51	18.50	27.65	40.33	31.15	19.26	22.13	27.46
Employment status	Subsistence farmers	28.87^**^	23.38	27.32	20.42	29.53^**^	22.79	24.41	23.27	47.91^**^	22.69	16.19	13.22
	Others	19.25	19.25	27.48	34.02	17.36	17.77	26.65	38.22	48.33	13.89	14.44	23.33
Marital status	Married	22.77^**^	21.65	27.84	27.74	24.39^**^	21.08	25.50	29.03	46.23^**^	21.34	16.27	16.16
	Others	36.52	21.35	24.72	17.42	35.56	22.22	22.22	20.00	57.86	20.75	13.84	7.55
Income	Q1 (~ 25%)	45.66^**^	21.86	21.22	11.25	40.38^**^	22.22	20.05	17.34	65.09^**^	18.18	12.00	4.73
	Q2	23.30	25.89	26.86	23.95	28.53	22.01	24.73	24.73	49.65	25.18	13.83	11.35
	Q3	16.61	20.27	30.56	32.56	19.47	20.43	25.96	34.13	42.52	21.65	17.32	18.50
	Q4(75%~)	12.94	19.09	30.74	37.22	16.09	19.84	29.22	34.85	34.19	19.85	20.59	25.37
Household composition	Parents and children	17.15^**^	20.84	25.07	36.94	17.37^**^	21.84	23.33	37.47	35.19^**^	17.59	18.52	28.70
	Three generations	20.16	19.75	28.40	31.69	21.67	20.00	29.05	29.29	36.97	20.61	18.79	23.64
	Elderly and children	25.00	20.83	31.94	22.22	30.00	17.69	20.00	32.31	44.08	27.63	15.13	13.16
	Only elderly	31.93	23.30	27.89	16.88	33.80	22.01	24.78	19.41	57.07	21.20	14.31	7.43
	Others	33.33	0.00	33.33	33.33	0.00	57.14	14.29	28.57	100.00	0.00	0.00	0.00
Medical insurance	NRCMs	24.68^**^	22.87	27.22	25.23	*26.05*	*20.84*	*25.03*	*28.08*	48.00^**^	21.40	15.91	14.70
	Other medical insurances	20.41	14.29	26.53	38.78	18.18	29.55	25.00	27.27	37.50	0.00	12.50	50.00
	No medical insurance	35.56	6.67	33.33	24.44	13.33	33.33	33.33	20.00	60.00	20.00	20.00	0.00
Social support	Q1 (~ 25%)	38.06^**^	21.67	24.44	15.83	35.42^**^	21.30	21.30	21.99	61.09^**^	19.94	10.93	8.04
	Q2	26.86	24.03	26.50	22.61	26.97	21.72	23.63	27.68	48.03	23.03	16.12	12.83
	Q3	18.55	22.01	29.56	29.87	20.96	20.40	25.21	33.43	42.11	26.32	16.12	15.46
	Q4(75%~)	12.68	18.66	29.58	39.08	16.52	21.32	31.83	30.33	34.32	11.24	24.26	30.18
Chronic disease	No	11.95^**^	17.99	31.12	38.94	11.58^**^	16.78	27.96	43.68	25.11^**^	22.49	24.24	28.17
	Yes	40.04	25.93	22.93	11.11	39.19	25.45	22.39	12.98	64.60	20.32	9.84	5.24
Clinic visit	No	13.29^**^	18.88	30.91	36.92	15.07^**^	18.65	28.13	38.15	24.19^**^	22.56	24.42	28.84
	Yes	40.46	25.43	22.93	11.18	42.96	25.60	19.93	11.51	63.88	20.16	10.39	5.58
Hospitalization	No	20.55^**^	22.11	28.99	28.35	22.63^**^	21.09	26.01	30.27	42.89^**^	21.98	18.40	16.73
	Yes	56.25	18.06	16.67	9.03	50.30	22.49	17.16	10.06	65.84	18.52	7.41	8.23
Active exercise	No	*25.30*	*21.38*	*28.38*	*24.94*	27.73^**^	21.04	24.47	26.75	*46.59*	*21.34*	*16.92*	*15.15*
	Yes	23.39	22.37	25.96	28.28	17.28	21.93	27.24	33.55	51.92	20.91	13.24	13.94
Smoke	No	26.27^**^	22.95	27.00	23.78	27.14^**^	21.78	24.54	26.55	50.17^**^	21.82	15.06	12.96
	Yes	19.12	16.91	29.41	34.56	20.30	19.70	26.57	33.43	37.29	18.08	20.34	24.29
Drink	No	27.25^**^	22.38	26.74	23.63	29.14^**^	21.07	24.13	25.66	50.43^**^	22.49	14.39	12.69
	< 3 times/week	16.48	20.88	19.78	42.86	8.27	20.30	27.07	44.36	35.71	10.71	28.57	25.00
	≥3 times/week	15.87	17.99	34.39	31.75	18.06	22.47	29.07	30.40	30.85	14.89	23.40	30.85
Region^**^		24.74	21.61	27.39	26.27	25.70	21.21	25.11	27.98	47.98	21.23	15.90	14.89

^*^significant difference among groups at *P* < 0.05 level; ^**^significant difference among groups at *P* < 0.01 level

Among all participants, the best levels of SRH among respondents aged 15 to 44 years were among participants who had higher levels of education, and were not subsistence farmers. Those who were married had higher SRH. Greater income also was associated with higher SRH. A total of 65.09% of the residents in the poorest income quartile in the northern region reported the poorest SRH. Elderly who lived alone reported the poorest.

### Strong social support positively impact good health

Among people in the northern region, those without medical insurance reported the poorest SRH (46.23%). However, medical insurance varied in the different regions. The residents who were covered by NRCMs exhibited the poorest SRH in the southern region (24.68%) and northern region (48.00%). However, the SRH of respondent with different medical insurance plans in the central region showed no statistical difference among them. Regardless of the region where the respondents resided, the entire sample with better social support had higher SRH scores. However, the trend was not consistent across the three regions; the highest SRH were found in quartile 4 in the southern and northern regions, and in quartile 3 in the central region.

### Better physical health consistent with better SRH

The participants with chronic illness, illness in the past two weeks, or a hospitalization within the past year, had poorer SRH than other participants. More than half reported the poorest SRH among respondents with chronic disease, had made a clinic visit and had been hospitalized in the northern region (64.60%, 63.88$ and 65.84%, respectively). However, the highest rate of poor SRH was among respondents with poor physical health in the southern and central regions.

### Health lifestyles positively impact good health

In the central region, the population who actively exercised had higher SRH than other participants (33.55%). In the southern and northern regions, the percentage of participants who exercised showed no statistical difference compared to the other respondents. However, most respondent who smoked or drank reported better SRH. These traits were more clearly represented in the southern and central regions; in the northern region, the highest rate of poor SRH was found among responders who did not smoke or drank.

Overall, the SRH among northern respondents was lower than that of southern and central regionals (*p* < 0.05), and nearly half of them (47.98%) reported the poorest SRH.

### Determinants of SRH in rural regions

The odds ratios of SRH in a series of models are presented in Table 4. In the first, districts were included, followed by a regression analysis of individual demographic characteristics, family factors, physical health and healthy lifestyle habits.

**Table 4:** Ordinal logistic regression of respondents’ SRH (Exp (B))

Variable	Model 1^a^	Model 2 ^b^	Model 3 ^c^	Model 4^d^	Model 5 ^e^	Model 6 ^f^
SRH(ref: Poorest)						
Poor	2.873^**^	4.967^**^	3.038^**^	2.433^**^	4.827^**^	3.447^**^
Good	1.331^*^	1.776^**^	1.061	0.844	1.479^*^	1.045
Best	0.384^**^	0.536^**^	0.315^**^	0.249^**^	0.387^**^	0.270^**^
Region (ref: Southern)						
Central	0.994	0.948	0.999	0.993	0.962	0.957
Northern	2.557^**^	2.139^**^	2.276^**^	2.278^**^	1.992^**^	1.932^**^
Gender (ref: Male)						
Female		1.184^*^	1.236^**^	1.255^**^	1.217^*^	0.879
Age (y) (ref: 15~)						
45~		1.825^**^	1.765^***^	1.782^**^	1.413^**^	1.472^**^
55~		2.663^**^	2.190^***^	2.155^**^	1.525^**^	1.562^**^
65~		3.899^**^	2.655^***^	2.578^**^	1.727^**^	1.772^**^
Education (ref: No education)						
Primary school		0.776^**^	0.811^**^	0.836^*^	0.896	0.859
Middle school or higher		0.501^**^	0.546^**^	0.576^**^	0.655^**^	0.661^**^
Income (ref: Q1)						
Q2			0.613^**^	0.647^**^	0.696^**^	0.706^**^
Q3			0.497	0.507^**^	0.555^**^	0.554^**^
Q4			0.447^**^	0.475^**^	0.498^**^	0.499^**^
Household composition (ref: Parents and children)						
Three generations				0.868	0.874	0.871
Elderly and children				0.784	0.775	0.767
Only elderly				1.118^*^	0.995^*^	0.988^*^
Others				2.114	2.032	2.291
Social support (ref: Poorer)						
Poor				0.792^**^	0.833^*^	0.620^**^
Good				0.701^**^	0.730^**^	0.735^**^
Better				0.608^**^	0.599^**^	0.836^*^
Chronic disease (ref: No)						
Yes					2.303^**^	2.235^**^
Clinic visit (ref: No)						
Yes					1.794^**^	1.807^**^
Hospitalization (ref: No)						
Yes					2.220^**^	2.126^**^
Smoke (ref: No)						
Yes						0.745^**^
Drink (ref: No)						
< 3 times/week						0.685^**^
≥3 times/week						0.627^**^
Chi-square	203.772	739.745	835.445	864.839	1374.133	1409.634
df	2	9	17	22	25	29
Sig.	0.000	0.000	0.000	0.000	0.000	0.000

ref: reference group; ^*^: *P* < 0.05; ^**^: *P* < 0.01

^a^Model 1 Single-factor analysis

^b^Model 2 Adjusted for individual traits (age, gender, education, employment)

^c^Model 3 Adjusted for the covariates in Model 2 and household characteristics (marital status, income, household composition)

^d^Model 4 Adjusted for the covariates in Model 3 as well as social support and medical insurance

^e^Model 5 All covariates in Model 4 and physical health (chronic illness, illness, hospitalization)

^f^ Model 6 All covariates in Model 5 and healthy habits (exercise, smoking and drinking)

Model 1 served to demonstrate that the place of residence was a significant determinant of poor SRH. The respondents who lived in the northern region were more likely to have poor SRH.

Model 2 indicated that gender, age and education were all associated with SRH. Female respondents were 1.184 times more likely than males to have poor SRH. The likelihood of poor SRH levels increased with age. Education demonstrated a protective effect; participants with higher level of education were less likely to have poor SRH.

As shown in the Model 3, the residents with higher incomes were less likely to have poor SRH than those who were impoverished. Participants in the wealthiest income quartile reported low SRH less than (0.447 times) those in the poorest quartile. However, marriage status, family composition and employment demonstrated no significant impact on SRH levels.

In Model 4, social support was included. The respondents who reported strong social support were 0.608 times more likely to have a poor SRH. Medical insurance was not included in the model, although the family composition was. The elderly living alone were more likely (1.118 times) to report poor SRH than the others.

Model 5 included the variables related to physical health: chronic diseases, illnesses, clinic visits and hospitalizations. These physical health variables were all significantly associated with SRH.

All of the covariates were analyzed in Model 6. The smoking population showed a lower likelihood (0.745 times) to report poor SRH. Furthermore, the people who drank more than twice a week were 0.627 times less likely to have poor SRH than people who did not drink. Respondents who actively exercised did not show lower SRH levels. Furthermore, gender was proved irrelevant in this model.

Logistic regression analysis revealed that region, age, education, household income, social support, physical health, healthy lifestyle habits were all associated with SRH.

## Discussion

In this study we investigated the association between five category factors and SRH, including individual demographic characteristics, family factors, social capital traits, physical health, and healthy lifestyle habits, using the data form a lower-middle-income rural society in China.

### Comparison to previous studies

The study’s findings factors of importance among regions with wide disparities, even within the same province. More importantly, the results suggested associations between SRH and a variety of factors, women, elderly, respondents with lower level of education, who lived alone, lower-income individuals, people with low levels of social support, and those with poor physical health all fared worse on SRH [10, 14, 19–21]. Additionally, smoking and consuming small amounts of alcohol were associated with good SRH.

### Region condition and residents’ SRH

Region of residence was found to affect SRH scores in all of the models. Previous studies have demonstrated that place of residence proved relevant to SRH scores [8, 22]. The findings form this study were consistent with those form previous studies, that suggested that household better economic conditions positively impacted SRH [15]. In this study, the northern region’s lower economic levels and decreased access to medical resources affected the quality of health services provided that, in turn, affected its residents’ reported health. Policymakers should pay attention to the northern residents’ relatively poor subjective health scores. Economic and public policies that address inequities in the quality of health care services provided may improve residents’ health [23].

This research demonstrated that marital status had no significant effect on SRH. Previous research also has shown that there were that no significant correlations between SRH and housing quality, or marital status [14]. The fact that the majority of the participants in this study were married may have contributed to this finding. High levels of social support corresponded to higher SRH scores. The measure of SRH permitted an individual to independently determine which factors contribute to their health. Rich social relationship networks could have contributed to positive perceptions about health, low levels of social support could be associated with low SRH [10, 24]. Perceptions about one’s own health could be enhanced through the creation of positive community environments and harmonious family atmospheres. The participants who experienced hypertension, diabetes, cancer or had been hospitalized were more likely to report poor health [17, 25]. The perception of a person’s overall health was affected by the appearance of chronic conditions or serious illnesses. Therefore, subjective perception also depended on objective health [5, 26].

### Medical insurance and SRH

This study evidenced no differences in SRH among residents with different types of medical insurance. This may have been because residents in these regions enjoyed coverage primarily through the NRCMs, with less access to other types of insurance. Since 2009, Anhui Province has gradually implemented a merger between NRCMs and urban resident medical insurance. The new insurance model will be referred to as “urban and rural resident medical insurance.” In some regions—particularly in the south—rural businesses have purchased private insurance or urban employee medical insurance for some part-time rural resident workers. The fact that some workers enjoyed double coverage could partially explain why medical insurance did not seem to impact SRH scores. This also demonstrated that access to additional types of insurance coverage (such as commercial medical insurance) and implementation of a more comprehensive medical insurance package could serve to improve rural residents’ perception of their health [22].

### Healthy lifestyle habits and SRH

The study sustained findings form previous research that better SRH was reported among people who smoked cigarettes and consumed alcohol [27]. However, this finding contradicted an earlier study that associated smoking and drinking with lower SRH scores [28] and another that found that the elderly with healthier lifestyles believed themselves to be healthier than those with less healthy lifestyles [29]. The participants in this study showed high incidence of unhealthy lifestyles; a little over one-fifth of them (20.39%) smoked. Interestingly, most of the smokers and drinkers were male and in good physical health. This could help to explain why smoking and drinking was associated with positive SRH. The possibility exists that the outcome resulted from the populations’ desire to believe that they were healthy despite their smoking and drinking habits. Furthermore, there could also be a cultural explanation for the association between smoking and drinking and good health. Both activities typically serve as a means to socialize. However, this does not in any way contradict the extensive research that has confirmed the harm caused by smoking or excessive drinking.

## Conclusion

Regional differences in SRH exist among the rural Chinese. Other factors also contribute to differences in SRH. After controlling for region, higher levels of household income, higher levels of education and good sources of social support improved SRH scores; being elderly, the presence of chronic illnesses, clinic visits and prior hospitalizations negatively impacted the scores.

Policy-makers should consider the benefits of programs when addressing health outcomes in financially distressed districts. Providing greater opportunities for economic development, strengthening social capital, increasing access to higher education and promoting to the availability of basic healthcare services could all serve to improve health outcomes, especially among the elderly and residents of low-income regions. Future research could further explore the positive impact of smoking and drinking on SRH.

### Limitations

This was cross-sectional study, no cause-effect relationship between each social factors and SRH could be determined.

## Abbreviations

NRCMs: New rural cooperative medical insurance
SRH: Self-rated health
SSRS: Social support rating scale
